# Prevalence and risk factors of violence against women and children during COVID-19, Germany

**DOI:** 10.2471/BLT.20.270983

**Published:** 2021-03-19

**Authors:** Cara Ebert, Janina I Steinert

**Affiliations:** a RWI - Leibniz Institute for Economic Research, Essen, Germany.; b Technical University of Munich, Richard-Wagner-Strasse 1, 80333 München, Germany.

## Abstract

**Objective:**

To assess the prevalence and exacerbating factors of violence against women and children in Germany during the coronavirus disease 2019 pandemic.

**Methods:**

We conducted a representative online survey with partnered women (18–65 years) between 22 April and 8 May 2020, when participants had been under lockdown for a month. We determined the prevalence of several forms of violence within the previous month using both direct elicitation and a list experiment. We conducted a multivariable logistic regression to assess the impact of pandemic-associated risk factors.

**Findings:**

Of our 3818 survey respondents, 118 (3.09%; 95% confidence interval, CI: 2.54 to 3.64) reported incidents of physical conflict, 293 (7.67%; 95% CI: 6.83 to 8.52) reported emotional abuse, and 97 (6.58%; 95% CI: 5.31 to 7.85) of 1474 respondents with children reported child corporal punishment. We estimated that 3.57% (95% CI: −0.33 to 7.46) had non-consensual intercourse with their partner. Our regression analysis revealed an increased risk of physical conflict with home quarantine (odds ratio, OR: 2.38; 95% CI: 1.56 to 3.61), financial worries (OR: 1.60; 95% CI: 0.98 to 2.61), poor mental health (OR: 3.41; 95% CI: 2.12 to 5.50) and young (< 10 years) children (OR: 2.48; 95% CI: 1.32 to 4.64); we obtained similar results for other forms of violence. Awareness and use of pertinent support services was low.

**Conclusion:**

Our findings of an increased risk of domestic violence during the pandemic should prompt policy-makers to improve the safety of women and children. Interventions to alleviate risks factors and extend support services are required.

## Résumé

**Objectif:**

Mesurer la fréquence et les facteurs aggravant la violence envers les femmes et les enfants en Allemagne durant la pandémie de maladie à coronavirus 2019.

**Méthodes:**

Nous avons mené une enquête représentative en ligne auprès de femmes vivant en couple (18–65 ans) entre le 22 avril et le 8 mai 2020, au moment où les participantes étaient confinées depuis un mois. Nous avons ensuite déterminé la fréquence de plusieurs formes de violence au cours du mois écoulé, en utilisant à la fois la technique d'élicitation directe et celle du «list experiment». Enfin, nous avons employé un modèle de régression logistique multivariée pour évaluer l'impact des facteurs de risque associés à la pandémie.

**Résultats:**

Sur nos 3818 participantes à l'enquête, 118 (3,09%; intervalle de confiance de 95%, IC: 2,54 à 3,64) ont signalé des actes de violence physique, 293 (7,67%; IC de 95%: 6,83 à 8,52) ont indiqué avoir subi des violences psychologiques et 97 (6,58%; IC de 95%: 5,31 à 7,85) parmi les 1474 participantes avec enfants ont rapporté des punitions corporelles sur les enfants. Selon nos estimations, 3,57% des participantes (IC de 95%: −0,33 à 7,46) ont eu des relations sexuelles non consenties avec leur conjoint. Notre analyse de régression a révélé que le risque de violences physiques augmentait en cas de quarantaine à domicile (odds ratio, OR: 2,38; IC de 95%: 1,56 à 3,61), de soucis financiers (OR: 1,60; IC de 95%: 0,98 à 2,61), de dégradation de la santé mentale (OR: 3,41; IC de 95%: 2,12 à 5,50) et en présence d'enfants de moins de 10 ans (OR: 2,48; IC de 95%: 1,32 à 4,64). Nous avons obtenu des résultats similaires pour d'autres formes de violence. Rares étaient celles qui avaient connaissance et faisaient appel aux services d'aide prévus à cet effet.

**Conclusion:**

Les résultats de notre enquête témoignent d’un risque accru de violence domestique durant la pandémie, et devraient encourager les législateurs à améliorer la sécurité des femmes et des enfants. Des interventions sont nécessaires pour atténuer les facteurs de risque et développer les services d'aide.

## Resumen

**Objetivo:**

Evaluar la prevalencia y los factores agravantes de la violencia contra las mujeres y los niños en Alemania durante la pandemia de la enfermedad del coronavirus 2019.

**Métodos:**

Realizamos una encuesta online representativa con mujeres en pareja (18-65 años) entre el 22 de abril y el 8 de mayo de 2020, cuando las participantes llevaban un mes confinadas. Determinamos la prevalencia de varias formas de violencia en el mes anterior utilizando tanto la elicitación directa como un experimento de lista. Realizamos una regresión logística multivariable para evaluar el impacto de los factores de riesgo asociados a la pandemia.

**Resultados:**

De las 3.818 encuestadas, 118 (3,09%; intervalo de confianza del 95%, IC: 2,54 a 3,64) informaron de incidentes de conflicto físico, 293 (7,67%; IC del 95%: 6,83 a 8,52) informaron de abuso emocional y 97 (6,58%; IC del 95%: 5,31 a 7,85) de los 1.474 encuestados con hijos informaron de castigos corporales infantiles. Se estimó que el 3,57% (IC 95%: -0,33 a 7,46) tuvo relaciones sexuales no consentidas con su pareja. Nuestro análisis de regresión reveló un mayor riesgo de conflicto físico durante la cuarentena (odds ratio, OR: 2,38; IC del 95%: 1,56 a 3,61), preocupaciones financieras (OR: 1,60; IC del 95%: 0,98 a 2,61), mala salud mental (OR: 3,41; IC del 95%: 2,12 a 5,50) y niños pequeños (< 10 años) (OR: 2,48; IC del 95%: 1,32 a 4,64); obtuvimos resultados similares para otras formas de violencia. El conocimiento y el uso de los servicios de apoyo pertinentes fueron bajos.

**Conclusión:**

Nuestros resultados sobre un mayor riesgo de violencia doméstica durante la pandemia deberían impulsar a los responsables políticos a mejorar la seguridad de las mujeres y los niños. Se requieren intervenciones para aliviar los factores de riesgo y ampliar los servicios de apoyo.

## ملخص

الغرض تقييم انتشار وعوامل تفاقم العنف ضد النساء والأطفال في ألمانيا خلال جائحة مرض فيروس كورونا 2019.

الطريقة أجرينا مسحًا تمثيليًا عبر الإنترنت مع نساء مشاركات (بسن 18 إلى 65 عامًا) بين 22 أبريل/نيسان و 8 مايو/أيار 2020، عندما كانت المشاركات في وضع الإغلاق لمدة شهر. قمنا بتحديد مدى انتشار أشكال عديدة من العنف خلال الشهر الماضي باستخدام كل من الاستنتاج المباشر وتجربة القائمة. قمنا بإجراء تحوف لوجيستي متعدد المتغيرات لتقييم تأثير عوامل الخطر المرتبطة بالجائحة.

**النتائج:**

من بين 3818 مشاركة في المسح، أبلغت 118 (3.09%؛ فاصل ثقة 95%: 2.54 إلى 3.64) عن حوادث للشجار البدني، وأبلغت 293 مشاركة (7.67%؛ فاصل ثقة 95%: 6.83 إلى 8.52) عن استغلال عاطفي، وأبلغت 97 من 1474 مشاركة (6.58%؛ فاصل ثقة 95%: 5.31 إلى 7.85) ممن لديهم أطفال عن عقاب جماعي للأطفال. قمنا بتقدير أن 3.57% (بفاصل ثقة 95%: -0.33 إلى 7.46) قد تعرضن لعلاقة جنسية دون رضا مع شركائهم. كشف تحليل التحوف لدينا عن زيادة خطر الشجار البدني في الحجر الصحي المنزلي (نسبة الأرجحية: 2.38؛ ‏بفاصل ثقة 95%: 1.56 إلى 3.61)، المخاوف المالية (نسبة الأرجحية: 1.60؛ ‏بفاصل ثقة 95%: 0.98 إلى 2.61)، الصحة العقلية الضعيفة (نسبة الأرجحية: 3.41؛ ‏بفاصل ثقة 95%: 2.12 إلى 5.50)؛ والأطفال الأصغر (أقل من 10 سنوات) (نسبة الأرجحية: 2.48؛ ‏بفاصل ثقة 95%: 1.32 إلى 4.64)؛ حصلنا على نتائج مماثلة لأشكال أخرى من العنف. كان الوعي واستخدام خدمات الدعم ذات الصلة منخفضًا.

الاستنتاج أن النتائج التي توصلنا إليها بخصوص زيادة مخاطر العنف المنزلي أثناء الجائحة، يجب أن تحث واضعي السياسات على تحسين أمان النساء والأطفال. هناك حاجة لتدخلات لتخفيف عوامل المخاطر وتمديد خدمات الدعم.

## 摘要

目的 旨在评估德国新型冠状病毒肺炎期间妇女儿童遭受暴力攻击的普遍性和相关刺激因素。

方法 在 2020 年 4 月 22 日至 2020 年 5 月 8 日期间，我们面向已居家隔离一个月之久的女性伴侣（18-65 岁）开展了一项具有代表性的线上调查。我们通过直接诱引和一系列实验，确定了前一个月内发生的几种普遍的暴力形式。我们进行了多变量逻辑回归分析，以评估大流行相关风险因素的影响。

结果 在 3818 名调查受访者中，118 名（3.09%；95% 置信区间，CI：2.54 至 3.64）报告存在肢体冲突，293 名（7.67％；95% CI：6.83 至 8.52）报告存在精神虐待，在 1474 名有子女的受访者中，97 名（6.58％；95% CI：5.31 至 7.85）报告存在体罚儿童。我们估计 3.57％（95% CI：-0.33 至 7.46）是在非自愿的情况下与其伴侣发生了性行为。根据我们的回归分析结果，因居家隔离（优势比，OR：2.38；95% CI：1.56 至 3.61）、财务担忧（OR：1.60；95% CI：0.98 至 2.61）、心理障碍（OR：3.41；95% CI：2.12 至 5.50）和年幼（<10 岁）儿童（OR：2.48；95% CI：1.3 至 4.64）导致发生肢体冲突的风险增加；基于其他暴力形式，我们得出了类似的结果。寻求相关支持服务的意识不强且此类服务的使用率很低。

结论根据我们的研究结果，大流行期间家庭暴力风险增加，决策者应加快提升妇女儿童的安全性。必要时进行干预，以降低风险并推广支持服务。

## Резюме

**Цель:**

Оценка распространенности насилия в отношении женщин и детей и усугубляющих его факторов в Германии во время пандемии коронавирусной инфекции 2019 года.

**Методы:**

Авторы провели репрезентативный интернет-опрос с участием женщин (18–65 лет) в период с 22 апреля по 8 мая 2020 года, когда участники находились в изоляции на протяжении месяца. Авторы установили распространенность нескольких форм насилия на протяжении предыдущего месяца, используя как методы прямого сбора информации, так и списочный эксперимент. Для оценки воздействия связанных с пандемией факторов риска использовалась многомерная логистическая регрессия.

**Результаты:**

Из 3818 респондентов опроса 118 (3,09%; 95%-й ДИ: 2,54–3,64) сообщали о случаях физического насилия, 293 респондента (7,67%; 95%-й ДИ: 6,83–8,52) сообщали об эмоциональном насилии и 97 из 1474 респондентов, имеющих детей, (6,58%; 95%-й ДИ: 5,31–7,85) сообщали о телесных наказаниях детей. Согласно проведенной авторами оценке 3,57% респондентов (95%-й ДИ: -0,33–7,46) имели половые отношения с партнером по принуждению. Регрессионный анализ выявил повышенный риск физического насилия в условиях домашнего карантина (показатель шансов, ПШ: 2,38; 95%-й ДИ: 1,56–3,61), финансовые проблемы (ПШ: 1,60; 95%-й ДИ: 0,98–2,61), слабое психическое здоровье (ПШ: 3,41; 95%-й ДИ: 2,12–5,50) и маленькие (< 10 лет) дети (ПШ: 2,48; 95%-й ДИ: 1,32–4,64); аналогичные результаты были получены в отношении других форм насилия. Уровень информированности и использования соответствующих вспомогательных служб был низким.

**Вывод:**

Полученные результаты о возросшем риске семейно-бытового насилия во время пандемии должны побудить лиц, ответственных за принятие стратегических решений, повысить уровень безопасности женщин и детей. Необходимы меры по уменьшению факторов риска и расширению спектра вспомогательных служб.

## Introduction

The World Health Organization (WHO) declared the coronavirus disease 2019 (COVID-19) outbreak a public health emergency of international concern on 30 January 2020.^1^ To curb the outbreak, many governments implemented social distancing interventions, such as school closures, requirements of working from home and restricting private contacts. Although social distancing regulations are necessary from a virological perspective, they may have unintended consequences and expose certain segments of the population to other physical and mental health risks. One of the most cited aspects in this regard is the rise in domestic violence against women and children.^2^^–^^8^ Empirical evidence from numerous countries, including Argentina, India, Peru and the United States of America, USA, has revealed an increase in the number of help requests to domestic abuse and child protection helplines during the pandemic.^9^^–^^12^ Further studies document a rise in domestic violence-related emergency calls to the police in several countries in the European Union and in Mexico, the United Kingdom of Great Britain and Northern Ireland and the USA,^13^^–^^17^ and higher admission numbers of abuse-related trauma patients in hospitals in South Africa and the United Kingdom.^18^^,^^19^

Systematic reviews and meta-analyses of prospective longitudinal studies have highlighted socioeconomic disadvantage, poor mental health, alcohol misuse by a partner, unplanned pregnancies and a history of childhood abuse as risk factors for domestic violence, while older age has been confirmed as a protective factor.^20^^–^^23^ In addition to these general risk factors, several COVID-19-specific mechanisms may increase the risk of domestic violence. First, home confinement can limit a person’s ability to escape potential perpetrators and seek social and professional support.^8^^,^^24^ Second, pandemic-induced economic pressures may exert a high level of financial distress and result in pecuniary losses for those on furlough or short-term work schemes, or who have become newly unemployed. Previous studies have revealed significant increases in domestic violence in the wake of economic recessions.^25^^–^^27^ Third, the closure of day care centres and schools inflicts a care burden on parents, causing them to renegotiate the distribution of household tasks, creating further potential for conflict.^28^ Lastly, social isolation, economic uncertainty and an increased care burden may have detrimental effects on mental health,^29^ a central risk factor for domestic violence in normal times.^30^^,^^31^

From a representative sample of women surveyed online, we aim to estimate the prevalence of violence against women and children in Germany during the COVID-19 pandemic. We also aim to determine the pandemic-related and general risk factors that contribute to an increased risk of some types of violence. 

## Methods

### Study design

We conducted our online survey between 22 April and 8 May 2020, when all states in Germany were enforcing strict policies to contain the spread of the pandemic. From 10 March 2020 onwards, schools, kindergartens, stores, restaurants and other public places were closed, and social contacts were limited to a minimum. We enrolled and interviewed 3818 partnered women aged 18–65 years (all of whom provided written electronic consent) via the survey firm respondi (repsondi, Köln, Germany), which offers a comprehensive participant pool of approximately 100 000 individuals. We applied quotas to ensure representativeness of respondents in terms of (i) German state, (ii) age, (iii) net household income, (iv) education, (v) employment status, and (vi) household size. To reduce the emotional burden for survivors when responding to violence-related questions, we used a small number of questions rather than the full WHO domestic violence questionnaire.^32^

In designing our study, we considered how domestic violence is a sensitive and stigmatized phenomenon and therefore prone to social desirability bias in self-reports.^33^^–^^35^ To tackle this issue, we adopted a two-pronged approach of (i) direct elicitation about less severe forms of violence, namely verbal and physical conflict with, or emotional abuse from, a partner, or corporal punishment of children; and (ii) indirect elicitation through double list experiments to measure sexual violence and more severe forms of physical violence against women and children. In single list experiments, respondents are randomly assigned to one of two lists; one list consists of four innocuous statements (reference group) and the other list includes these same four statements plus an additional sensitive item (experimental group). Respondents are then asked to specify the number of presented statements that apply to them (e.g. “3 out of 5”), allowing the researcher to establish the prevalence of violence by comparing the average total number between the reference and the experimental group. To increase statistical power,^36^ we employed a double list experiment in which all respondents were presented with two distinct lists per outcome of interest, one with and one without the sensitive item. 

Our survey took 15–20 minutes to complete, and respondents received a small financial incentive (an online shopping voucher) to participate. Since face-to-face debriefings after completion of the interview were not possible, we provided respondents with information about selected domestic abuse helplines and email contacts (see details in data repository).^37^

### Study data

Wherever possible, we adapted measures of violence from previous surveys conducted in Germany to ensure contextual relevance. We piloted individual violence questions and constructed list experiments in waves 6 and 12 of the German COVID-19 Snapshot Monitoring study,^38^ and cross-correlated these with other measures of violence as well as presumed predictors for validation purposes. We also included a social desirability scale validated in the German context to assess its effect on the reporting rates of violence.^39^ We provide all constructed variables in the online data repository.^37^

Our survey also elicited information on COVID-19-specific stressors that may exacerbate violence risk. We used the validated short version of the depression and anxiety scale (Patient Health Questionnaire 4) to capture the current mental health status of respondents and their partners.^40^ We included additional items to capture the more direct mental health impacts of the pandemic, such as physical anxiety symptoms linked to COVID-19 fears. Because we only interviewed women, we asked respondents to provide an assessment of their partner’s mental health status. We captured financial distress through an adapted list of questions used previously in Australia,^41^ as well as through reported actual financial losses as a result of the pandemic. We recorded whether respondents had been under home quarantine and, to assess the increased childcare burden caused by closures of day care centres and schools, we enquired about the age of children in the household and hours spent on childcare. Finally, we determined the awareness and use of existing support services for survivors of domestic violence in Germany.

### Statistical analysis 

We aggregated measurement instruments of mental health, financial concerns and social desirability into continuous-scale scores by using principal component analysis to weight individual items. All scales showed good internal consistency (we obtained Cronbach’s *α* of 0.81, 0.83, 0.84 and 0.69 for respondents’ mental health, partners’ mental health, financial concerns and social desirability, respectively). 

We tested hypothesized risk factors using a multivariable logistic regression model and individual-level binary outcome variables from direct elicitation (i.e. less severe forms of violence). We included general predictors of domestic violence, such as women’s age and socioeconomic status (as captured by household income, level of education and employment status of both respondent and partner before the pandemic), in our model. We also controlled for household size, whether partners cohabitated and whether the respondent was employed in the health sector or other essential services. In cases where the respondent was not able to evaluate her partner’s mental health, we substituted missing values using multiple imputation by chained equations.^42^

We conducted all our statistical analyses using the software Stata version 16.0 (StataCorp, College Station, USA).

### Ethics

Our study was approved by the ethics committee of the medical faculty at the Technical University of Munich (TUM, IRB 227/20 S).

## Results

### Study population

We summarize the sociodemographic characteristics of our study population in Table 1. Of our 3818 study participants, 657 (17.21%) reported having been home-quarantined, 91 (2.38%) reported that either they or their partner had lost their job and 1091 (28.58%) were subjected to short-term work or furlough as a result of the pandemic. We observed that 716 (18.75%) respondents revealed worries about their own or their partner’s job security, 380 (9.95%) were flagged for potential depression and 108 (2.83%) reported that thinking about the pandemic elicited immediate physical reactions.

**Table 1:** Characteristics of women included in online survey to assess prevalence and factors of violence against women and children during the coronavirus disease 2019 pandemic, Germany, April–May 2020

Sociodemographic characteristics (*n* = 3818)	No. women (%)
**Cohabitating**	3474 (90.99)
**Born abroad (or parents born abroad)**	562 (14.72)
**With** ≥ **4 members of household**	925 (24.23)
**With children < 10 years **	972 (25.46)
**With children **≥ **10 years**	718 (18.81)
**Net household income before pandemic (€)**	
< 2000	756 (19.80)
2000–4000	1635 (42.82)
> 4000	925 (24.23)
Prefer not to say	502 (13.15)
**Education **	
Middle school or less	1269 (33.24)
Lower secondary	1074 (28.13)
Higher secondary or more	1475 (38.63)
**Employed (Feb 2020)**	2842 (74.44)
**Employed as key worker**	1007 (26.38)
**Partner employed (April 2020)**	3000 (78.58)
**Under home quarantine**	657 (17.21)
**Financial impact of pandemic **	
Actual unemployment (woman or partner)	91 (2.38)
Reduced employment or furlough (woman or partner)	1091 (28.58)
**Financial worries**	
Unemployment (own or partner)	716 (18.75)
Insufficient income	448 (11.73)
**Mental health**	
Sad most days	380 (9.95)
Anxious about the pandemic	108 (2.83)
**Partner’s mental health (as assessed by respondent)^a^**	
Sad most days	353 (9.70)
Anxious about the pandemic	97 (2.67)
**Region**	
North	602 (15.77)
East	755 (19.77)
West	1375 (36.01)
South	1086 (28.44)

€: euros.

^a^ We only had complete data on partner’s mental health for 3638 of the respondents.

Note: We included women aged 18–65 years (mean: 44.30; standard deviation: 12.02) in the study.

### Prevalence of violence

Of our study population, a total of 967 (25.33%; 95% confidence interval, CI: 23.95 to 26.71) and 118 (3.09%; 95% CI: 2.54 to 3.64) women reported verbal and physical conflict, respectively, with their partner during the previous month. We noted that women were also exposed to emotional forms of abuse: 146 (3.82%; 95% CI: 3.22 to 4.43) indicated that they felt threatened by their partner; 85 (2.23%; 95% CI: 1.76 to 2.69) were confined within their homes; and 175 (4.58%; 95% CI: 3.92 to 5.25) reported being controlled in terms of restricted communication with contacts outside their homes. We learned that 97 of 1474 women (6.58%; 95% CI: 5.31 to 7.85) or another household member had corporally punished (one of) their children in the past month (Table 2). The number of respondents who experienced multiple forms of violence is reported in the data repository.^37^

**Table 2:** Estimated prevalence of violence against women and children during coronavirus disease 2019 pandemic, Germany, April–May 2020

Method of elicitation and type of violence	*n* = 3818	
	No. women	Estimated prevalence (95% CI)^a^
**Direct elicitation**		
Verbal conflict	967	25.33 (23.95 to 26.71)
Physical conflict	118	3.09 (2.54 to 3.64)
Emotional abuse (any)	293	7.67 (6.83 to 8.52)
Threatened	146	3.82 (3.22 to 4.43)
Confined	85	2.23 (1.76 to 2.69)
Controlled	175	4.58 (3.92 to 5.25)
Corporal punishment of children^b^	97	6.58 (5.31 to 7.85)
**Indirect elicitation^c^**		
Physical violence	NA	1.53 (−2.05 to 5.11)
Sexual violence	NA	3.57 (−0.33 to 7.46)
Physical violence against children^b^	NA	1.97 (−4.23 to 8.18)

CI: confidence interval; NA: not applicable.

^a^ For direct elicitation, the prevalence estimates indicate the calculated percentage. For indirect elicitation, the prevalence estimates are an average of the differences in the number of applicable statements between reference and experimental groups.

^b^ The sample size (*n*) for physical violence against children was 1474.

^c^ For indirect elicitation, we did not observe any individual-level experience of violence and therefore cannot observe the number of cases.

**Fig. 1:**
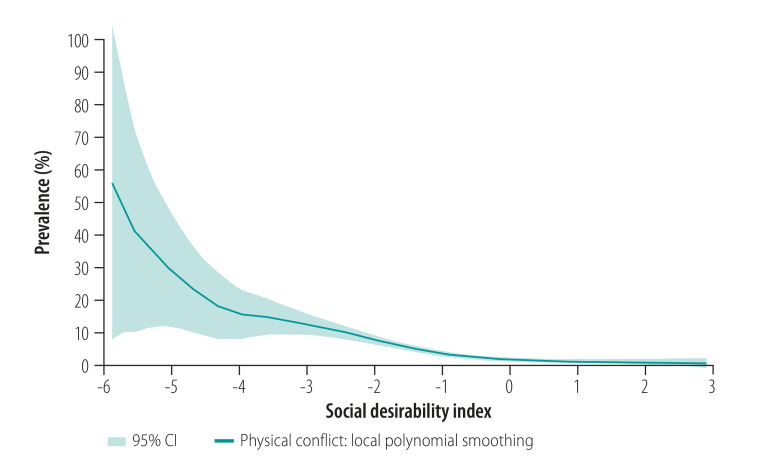
Negative association between reported prevalence of physical conflict and social desirability in study of violence against women and children during the coronavirus disease 2019 pandemic, Germany, April–May 2020

The real prevalence of violence was likely underestimated due to misreporting and social desirability bias. The negative association between respondents’ sensitivity to social desirability and disclosures of violence depicted in Fig. 1 and in the data repository^37^ corroborates this.

CI: confidence interval.

Note: Figure shows the local polynomial smoothing of degree 1 using the Epanechnikov kernel function of the binary variable physical conflict with partner on a continuous index of social desirability.

Based on double list experiment elicitation, we estimated that during the previous month the prevalence of physical violence was 1.53% (95% CI: −2.05 to 5.11), for non-consensual sex it was 3.57% (95% CI: −0.33 to 7.46) and for violence against children it was 1.97% (95% CI: −4.23 to 8.18) (Table 2).

### Risk factors

Compared with households not under quarantine, the risk of physical conflict was more than double in households under home quarantine (odds ratio, OR: 2.38; 95% CI: 1.56 to 3.61). Quarantine was also associated with a significantly higher risk of experiencing emotional abuse during the previous month in terms of being confined to the home (OR: 2.80; 95% CI: 1.70 to 4.60) or controlled (OR: 2.52; 95% CI: 1.79 to 3.54; Table 3). 

**Table 3:** Risk factors associated with less severe types of violence against women and children during coronavirus disease 2019 pandemic, Germany, April–May 2020

Risk factors	Odds ratio (95% CI)^a^						
	Women (*n* = 3638)						Children (*n* = 1416)
	Verbal conflict	Physical conflict	Emotional abuse				Corporal punishment
			Threatened	Confined	Controlled		
**Related to pandemic**							
Under home quarantine	1.10 (0.89 to 1.35)	2.38 (1.56 to 3.61)	1.43 (0.95 to 2.16)	2.80 (1.70 to 4.60)	2.52 (1.79 to 3.54)		1.31 (0.79 to 2.16)
Financial worries	0.95 (0.76 to 1.18)	1.60 (0.98 to 2.61)	1.58 (1.01 to 2.46)	1.29 (0.74 to 2.25)	1.22 (0.83 to 1.80)		0.81 (0.46 to 1.40)
Newly unemployed or reduced work	1.12 (0.90 to 1.38)	2.07 (1.23 to 3.49)	1.17 (0.70 to 1.95)	0.92 (0.49 to 1.73)	1.17 (0.75 to 1.81)		1.54 (0.86 to 2.76)
Partner newly unemployed or reduced work	1.18 (0.96 to 1.44)	1.07 (0.66 to 1.73)	1.02 (0.66 to 1.60)	1.25 (0.69 to 2.25)	0.84 (0.55 to 1.29)		0.98 (0.55 to 1.73)
With poor mental health	1.97 (1.60 to 2.43)	3.41 (2.12 to 5.50)	2.97 (1.91 to 4.62)	1.98 (1.16 to 3.39)	1.46 (1.00 to 2.13)		2.07 (1.17 to 3.64)
Partner with poor mental health^b^	2.17 (1.76 to 2.68)	2.23 (1.36 to 3.65)	3.82 (2.45 to 5.96)	4.12 (2.38 to 7.12)	2.82 (1.95 to 4.08)		2.71 (1.54 to 4.76)
Hours spent on child care^c^	1.07 (1.02 to 1.12)	1.01 (0.91 to 1.13)	1.06 (0.96 to 1.17)	0.96 (0.83 to 1.10)	1.00 (0.92 to 1.10)		0.97 (0.89 to 1.06)
With children < 10 years^d^	1.44 (1.08 to 1.93)	2.48 (1.32 to 4.64)	1.47 (0.79 to 2.76)	2.23 (0.99 to 5.01)	1.84 (1.04 to 3.25)		5.31 (2.16 to 13.03)
With children ≥ 10 years^d^	0.94 (0.73 to 1.22)	1.36 (0.75 to 2.48)	1.22 (0.72 to 2.06)	1.01 (0.54 to 1.90)	1.13 (0.72 to 1.77)		1.0 (–)
**General**							
Age (years)^c^	0.97 (0.96 to 0.98)	0.97 (0.95 to 0.99)	0.98 (0.96 to 1.00)	0.99 (0.97 to 1.01)	0.98 (0.96 to 1.00)		0.99 (0.96 to 1.02)
Education							
Middle school or less	1.0 (–)	1.0 (–)	1.0 (–)	1.0 (–)	1.0 (–)		1.0 (–)
Lower secondary	1.18 (0.95 to 1.47)	1.25 (0.72 to 2.15)	0.91 (0.56 to 1.47)	1.28 (0.70 to 2.33)	1.01 (0.68 to 1.52)		1.93 (1.02 to 3.68)
Higher secondary or more	1.45 (1.15 to 1.82)	0.97 (0.54 to 1.75)	0.69 (0.42 to 1.15)	0.77 (0.40 to 1.50)	0.63 (0.40 to 1.00)		2.08 (1.06 to 4.05)
Household income (net) before pandemic (€)							
< 2000	1.0 (–)	1.0 (–)	1.0 (–)	1.0 (–)	1.0 (–)		1.0 (–)
2000–4000	0.96 (0.76 to 1.21)	0.66 (0.40 to 1.10)	0.90 (0.56 to 1.47)	0.49 (0.27 to 0.89)	0.71 (0.47 to 1.08)		0.51 (0.29 to 0.89)
> 4000	0.99 (0.75 to 1.32)	0.21 (0.08 to 0.55)	0.61 (0.30 to 1.26)	0.50 (0.22 to 1.14)	0.42 (0.23 to 0.77)		0.37 (0.18 to 0.76)
Prefer not to say	0.86 (0.63 to 1.17)	0.58 (0.25 to 1.34)	0.61 (0.27 to 1.38)	0.15 (0.04 to 0.64)	0.35 (0.16 to 0.75)		0.47 (0.18 to 1.21)
Employed (Feb 2020)	1.17 (0.92 to 1.48)	0.77 (0.40 to 1.49)	0.80 (0.45 to 1.41)	0.66 (0.34 to 1.30)	1.18 (0.74 to 1.88)		0.89 (0.44 to 1.81)
Partner employed (Feb 2020)	0.77 (0.61 to 0.97)	0.71 (0.40 to 1.25)	0.70 (0.42 to 1.15)	1.32 (0.65 to 2.70)	0.58 (0.38 to 0.89)		1.15 (0.49 to 2.71)
**Other factors**							
Employed as key worker	1.06 (0.88 to 1.29)	2.35 (1.45 to 3.82)	1.74 (1.11 to 2.73)	1.75 (1.00 to 3.07)	1.36 (0.93 to 1.99)		1.12 (0.68 to 1.86)
No. members of household^c^	0.98 (0.87 to 1.11)	0.88 (0.68 to 1.15)	0.97 (0.77 to 1.23)	1.04 (0.79 to 1.36)	1.15 (0.96 to 1.39)		1.16 (0.92 to 1.46)
Cohabitating	1.31 (0.96 to 1.79)	1.39 (0.69 to 2.82)	1.04 (0.55 to 1.98)	1.15 (0.51 to 2.58)	0.69 (0.40 to 1.19)		0.95 (0.35 to 2.63)

CI: confidence interval; €: euros.

^a^ Standard errors are heteroskedasticity robust.

^b^ We only had complete data on partner’s mental health for 3638 of the respondents. We substituted missing values by multiple imputation via chained equations,^42^ based on responses to other questions related to depression and anxiety.

^c^ Continuous risk variables, for which the increased risk relates to one additional unit.

^d^ Compared to households with no children.

We noted an increased risk of some forms of violence for respondents in the highest quintile of the financial concerns scale, in terms of both physical conflict (OR: 1.60; 95% CI: 0.98 to 2.61) and feeling threatened (OR: 1.58; 95% CI: 1.01 to 2.46). Women in the highest quintile of the depression and anxiety scale were more likely to report the occurrence of verbal (OR: 1.97; 95% CI: 1.60 to 2.43) and physical (OR: 3.41; 95% CI: 2.12 to 5.50) conflict with their partner, all types of emotional abuse, as well as occurrences of child corporal punishment in the previous month (OR: 2.07; 95% CI: 1.17 to 3.64; Table 3). 

Similarly, we estimated a higher risk of violence with increased depression and anxiety in partners, with ORs of 2.23 (95% CI: 1.36 to 3.65) for physical conflict, 2.71 (95% CI: 1.54 to 4.76) for corporal punishment of children, and 2.82 (95% CI: 1.95 to 4.08) to 4.12 (95% CI: 2.38 to 7.12) for emotional abuse (Table 3). 

Apart from a significantly higher odds of verbal conflict (OR: 1.07; 95% CI: 1.02 to 1.12), we found no association between the daily childcare burden, measured in hours spent on caregiving per day, and risk of violence. Strikingly, we discovered that the presence of young children (< 10 years) in the home is a risk factor. Compared with households without young children, the risk of child corporal punishment quintupled in families with one or more young children (OR: 5.31; 95% CI: 2.16 to 13.03). We also noted the increased risks of verbal and physical conflict, with ORs of 1.44 (95% CI: 1.08 to 1.93) and 2.48 (95% CI: 1.32 to 4.64), respectively, in households with young children. The risk of emotional forms of violence was also significantly increased; for example, the OR of being confined to the home was 2.23 (95% CI: 0.99 to 5.01; Table 3). In robustness checks, we obtained similar results when including the social desirability bias index as an additional control and when estimating risk from only pandemic-specific or general factors (see tables in data repository).^37^

**Fig. 2:**
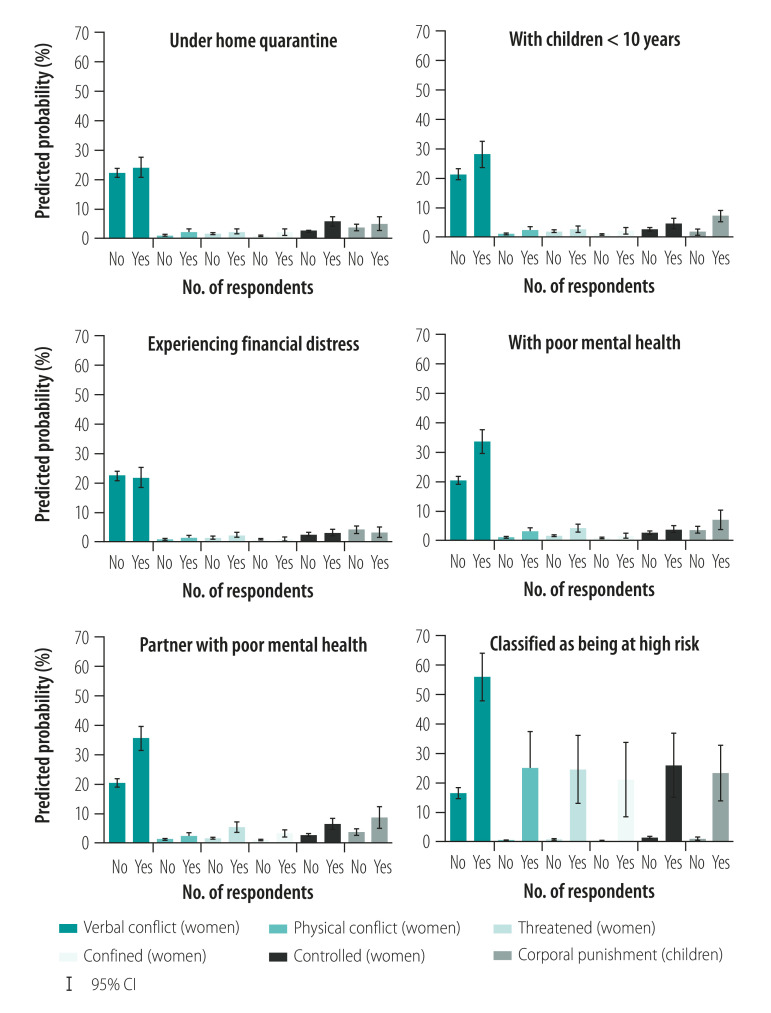
Predicted probability of violence for different risk factors in study of violence against women and children during the coronavirus disease 2019 pandemic, Germany, April–May 2020

We predicted probabilities of less severe forms of violence by risk factor, while holding all other risk factors and covariates constant at means (Fig. 2 and estimated prevalence of violence by risk factors measured on continuous scales in the data repository).^37^ As shown in Fig. 2, we predict the probability of violence for women with hypothetical high-risk (Yes) and low-risk (No) profiles. The predicted probability of emotional and physical forms of violence was almost zero in the low-risk scenario. In the high-risk scenario, we calculated predicted probabilities of 25.17% (95% CI: 12.73 to 37.61) for physical conflict, 21.12% (95% CI: 8.46 to 33.79) to 26.02% (95% CI: 14.94 to 37.10) for emotional abuse, and 23.32% (95% CI: 13.79 to 32.85) for child corporal punishment. We observed similar patterns of risk factors for more severe forms of violence as elicited in the list experiments (data repository).^37^

CI: confidence interval.

Notes: All risk factors applicable are included in the panel classified as being at high risk. Bar charts of predicted probabilities of verbal conflict, physical conflict, feeling threatened, being confined and being controlled and of violence against children based on the multivariable logistic regression estimates.

We further investigated the effect of young children in the home separately for women who either worked full-time, part-time or not at all in February 2020 (data repository).^37^ The presence of young children was significantly associated with an increased risk of physical conflict and emotional abuse for women working full-time only, and with an increased risk of corporal punishment of children for women working full-time and part-time.

**Fig. 3:**
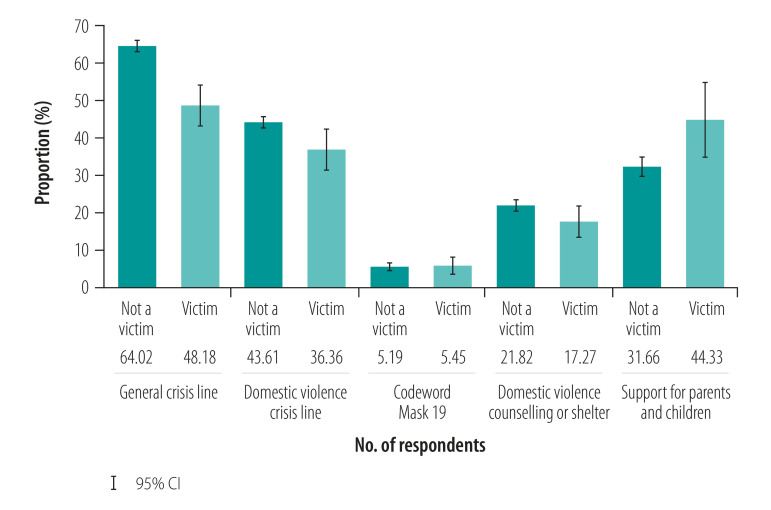
Awareness of help services in study of violence against women and children during the coronavirus disease 2019 pandemic, Germany, April–May 2020

**Fig. 4:**
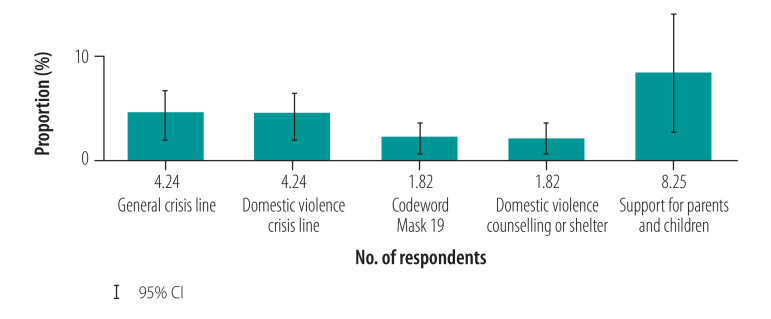
Use of help services in study of violence against women and children during the coronavirus disease 2019 pandemic, Germany, April–May 2020

We discovered that awareness of domestic violence help services was generally low (Fig. 3). Likewise, utilization of support services among violence survivors was low (Fig. 4), ranging from 1.82% (95% CI: 0.37 to 3.27) for both counselling centres and the codeword “Mask 19” in pharmacies to 8.25% (95% CI: 2.67 to 13.82) for the parenting crisis line among women who reported the occurrence of violence against children in their homes.

CI: confidence interval.

Note: We defined a victim as women who have been exposed to at least one form of violence in the past four weeks. The sample size for respondents classified as not being a victim was 3488, except for the category Support for parents and children, which was 1377. The sample size for respondents classified as being a victim was 330, except for the category Support for parents and children, which was 97.

CI: confidence interval.

Note: Restricted to women who experienced any form of conflict or violence with their partner (four left bars) or women with children in the household who experienced violence (far right bar). The sample size for respondents was 330, except for the category Support for parents and children, which was 97.

## Discussion

Our survey-based data have the advantage of being more suitable than administrative data for establishing the effect of the pandemic on the prevalence of domestic violence.^29^^,^^43^ Our findings of an increased risk of violence with pandemic-induced financial worries or poor mental health of either the respondent or her partner are confirmed by other studies: a survey conducted in Canada during the first COVID-19 lockdown found higher levels of violence among families who were unable to meet current financial obligations,^28^ and a survey conducted in the USA in spring 2020 found that parents who had reported depression and anxiety symptoms within the previous two weeks exhibited a greater potential for child abuse.^44^ We also observed that one of the most pronounced risk factors was the presence of young children in the home, corroborating a study based in the USA that showed the highest increase in calls to domestic violence helplines from such households.^12^

Our observation that the risks of violence and conflict were higher during phases of home quarantine was verified by a study from Argentina, which found a lower prevalence of violence among women whose partners did not have to comply with a stay-at-home order.^11^ Quarantine orders increase the time that partners spend together, often in the context of additional pressures such as childcare responsibilities, limited physical space and isolation from support networks outside the home. While the criminological theory of exposure reduction between intimates predicts a decline in violence, physical distancing regulations mechanically increase exposure between partners and thus violence risk.^28^^,^^45^

We found that women affected by violence were underutilizing the available support infrastructure. Potential barriers to accessing help services could include perceived stigma or a lack of privacy at home, particularly in the presence of a perpetrator.^46^ In addition, some women might be less comfortable with email counselling than in-person counselling, and were therefore alienated from support services as a result of the pandemic. In contrast, we found that awareness and use of help services for children at risk of violence was higher; this could imply that the stigma of seeking support is lower when women are not victims themselves.

Our study had several limitations. First, the cross-sectional design of our study meant that we were unable to establish reliable estimates of the extent to which domestic violence has increased as a direct consequence of the pandemic’s physical distancing laws. Comparisons with prevalence estimates from surveys conducted before the pandemic are inadequate, as these rely on previous-year or lifetime experiences rather than the past month. Second, the risk factors that we discuss are not causally identified and should therefore be interpreted as associations. This is particularly the case for mental health, which could be both a cause and a consequence of domestic violence. To partly address potential confounding, we controlled for a large number of arguably relevant and simultaneously operating factors. Third, although online surveys have several advantages, such as increased anonymity, they can be prone to selection bias.^47^^,^^48^ For example, women with controlling partners might have had difficulties participating in the survey. However, we expect that the association between risk factors and violence is less affected by potential selection bias than the prevalence estimate itself. Fourth, the benefits of the double list experiment in terms of violence disclosures and respondent protection come at the cost of reduced statistical efficiency; results obtained via indirect elicitation were therefore excluded from the regression analysis of risk factors. While we sought to account for possible underreporting of violence by using list experiments and social desirability controls, disclosures may still have been inhibited by fear of reprisal^49^ or by post-traumatic amnesia.^50^ Fifth, we relied on respondents for an assessment of the mental health status of their partners. However, survivors of violence might perceive their partners’ mental condition more negatively than women who are not exposed to violence, which may lead to an upward bias of the coefficient.

Our findings of an increased risk of domestic violence in times of crises should prompt policy-makers to improve the safety of women and children. In anticipation of future lockdowns in Germany and other countries, interventions to alleviate risk factors and extend support services for survivors of violence – including emergency childcare centres to reduce parental stress, state-provided financial relief packages to reduce financial concerns, and the provision of easily accessible phone- and internet-based mental health counselling – are urgently required. 
